# A Rare Case of a Giant Sialolith Within Wharton’s Duct

**DOI:** 10.7759/cureus.35969

**Published:** 2023-03-10

**Authors:** Konstantinos Chaidas, Stergios Lialiaris, Angeliki Vasiliki Pavlou, Michael Katotomichelakis, Sotirios Papouliakos

**Affiliations:** 1 Ear, Nose, and Throat Department, University Hospital of Alexandroupolis, School of Medicine - Democritus University of Thrace, Alexandroupolis, GRC; 2 Ear, Nose, and Throat Department, General Hospital of Athens “G. Gennimatas”, Athens, GRC

**Keywords:** case report, surgical management, megalith, wharton’s duct, giant sialolith, sialolithiasis

## Abstract

Sialolithiasis is a common disease characterized by the formation of calculi within the salivary glands or their ducts. Although many cases of large stones located within the submandibular gland have previously been reported, the presence of a giant stone within Wharton’s duct is extremely rare. We report the case of a patient who presented with an unusually large stone measuring about 6 cm in the greatest dimension located within Wharton’s duct and causing local swelling and pain. The sialolith was successfully removed intraorally indicating that a minor procedure under local anesthesia can be a successful treatment modality even in the case of a giant sialolith.

## Introduction

Sialolithiasis is a situation concerning the formation of stones in the salivary glands or within their ducts. It is the most common cause of salivary gland swelling with an occurrence of 1 in 10,000 to 1 in 30,000 cases [1]. A sialolith can lead to obstruction of the salivary gland and local inflammation as a result of bacterial infection. The diagnosis can easily be made by detailed history and clinical examination, especially in the presence of a large sialolith, but imaging such as ultrasound scan, computed tomography scan, magnetic resonance imaging scan, and sialoendoscopy can be helpful, especially in small stones that are difficult to appreciate on palpation. There are various treatment options available, including conservative and/or surgical methods. Here, we present an extremely rare case of a giant sialolith located within Wharton’s duct, which was successfully managed with intraoral removal under local anesthesia.

## Case presentation

A 72-year-old male patient presented at the Ear, Nose, and Throat Emergency Clinic due to gradually worsening right submandibular swelling with an acute onset a week ago. He also complained of local pain, tenderness, and mild odynophagia. There was no fever, breathing difficulties, or other significant relevant medical history.

On clinical examination, mild restriction of mouth opening was noted. There was a visible swelling in the anterior part of the right side of the floor of the mouth. Throughout the palpation of the area, a large, hard mass was perceived across Wharton’s duct. The right submandibular gland was enlarged, tender, and painful on palpation. There was an absence of salivary flow due to complete obstruction of the duct orifice. Flexible nasoendoscopy revealed a patent upper airway with only mild edema of the right-sided pharyngeal wall.

**Figure 1:**
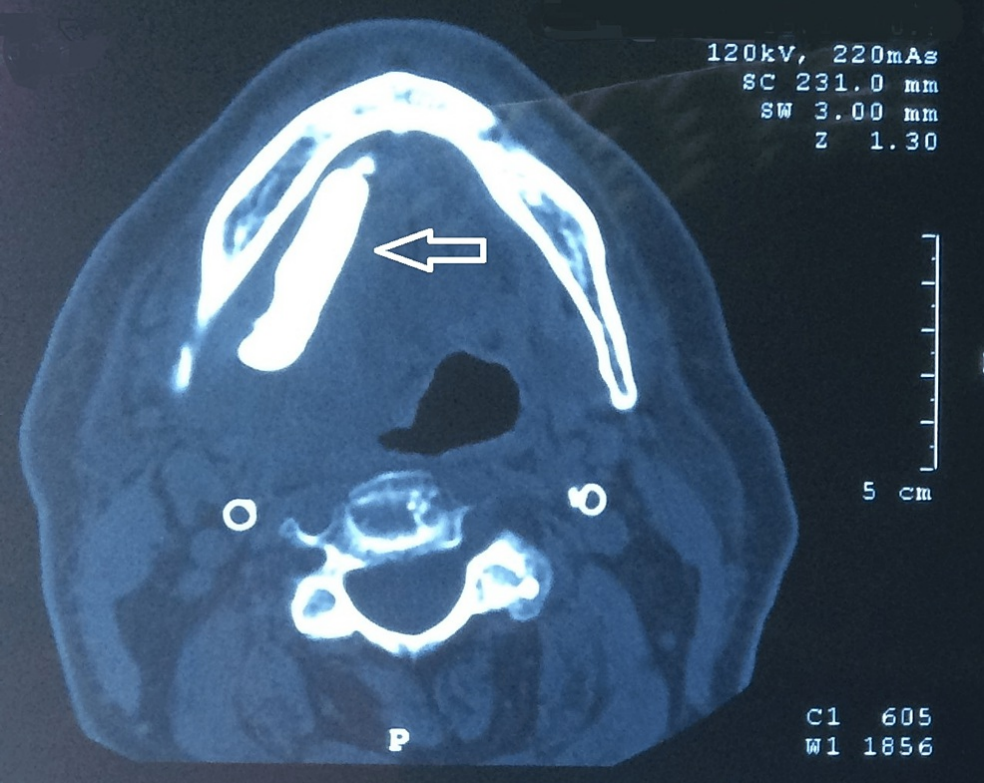
Computed tomography shows the presence of a giant stone in the duct of the right submandibular gland (white arrow).

The computed tomography (CT) scan showed the presence of an elongated radio-opaque shadow with clear boundaries in the course of the right submandibular gland duct (Figure 1).

There was also swelling in the right submandibular region with diffused margins. Mild right-sided level IIa and IIb cervical lymphadenopathy was also noticed.

**Figure 2:**
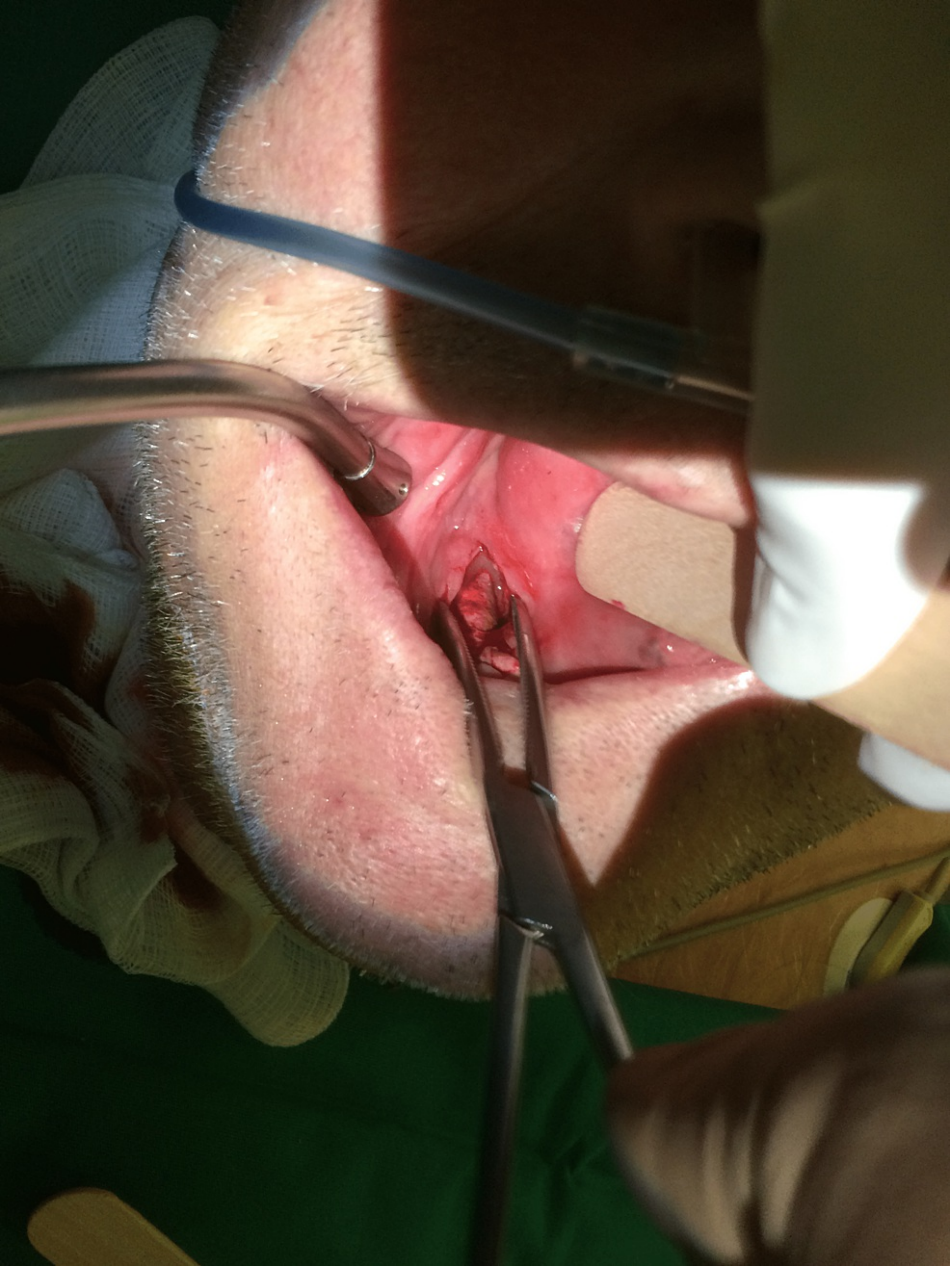
Intraoperative photograph showing the transoral removal of the sialolith.

**Figure 3:**
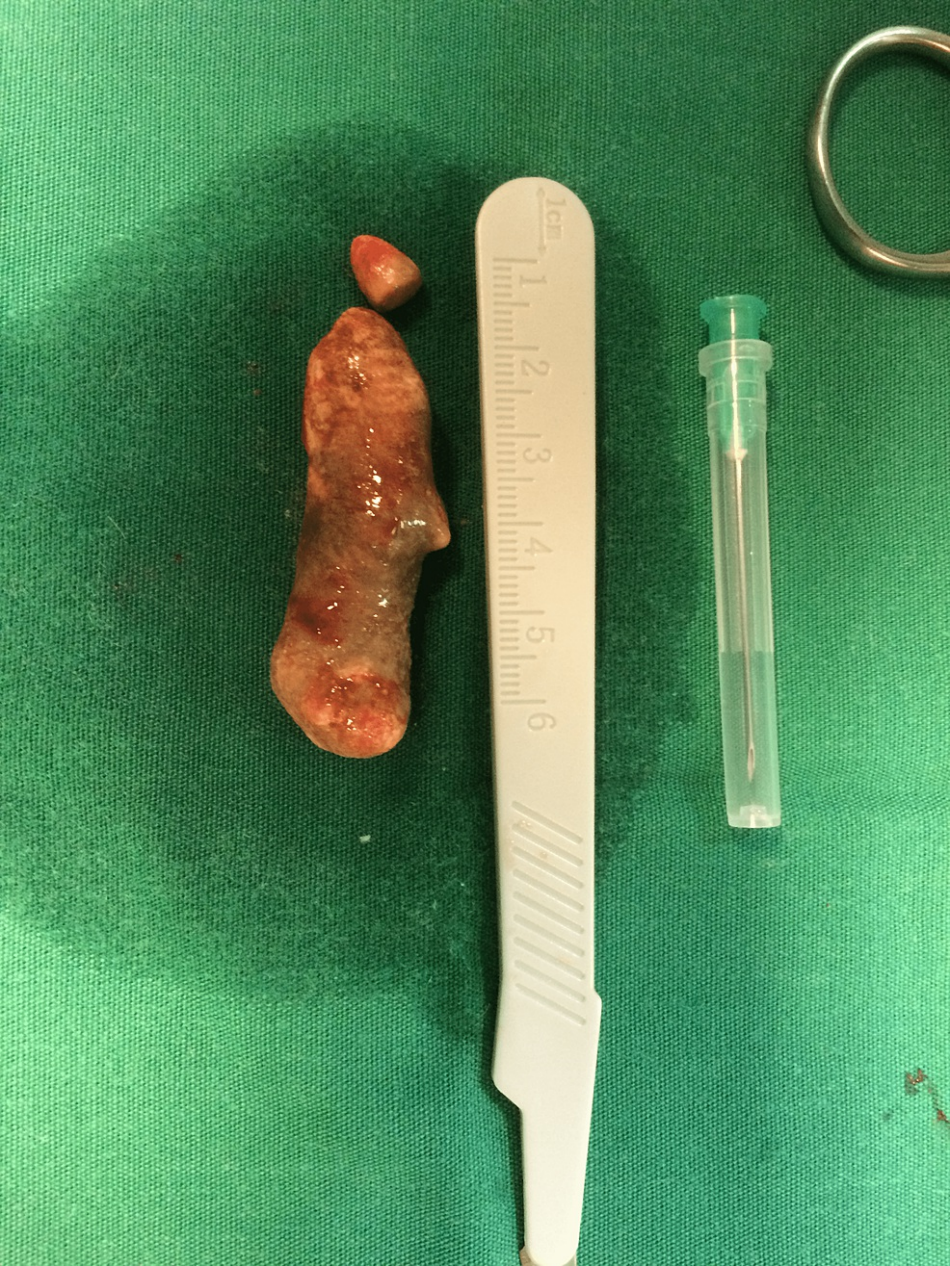
Giant sialolith (in two pieces) measuring 58 mm × 17 mm.

The findings were strongly suggestive of an extreme form of sialolithiasis in the right Wharton’s duct with secondary submandibular gland inflammation and associated lymphadenopathy. The patient underwent intraoral removal of the unusually large stone via open surgery under local anesthesia (Figures 2, 3). An incision over the palpated stone and across the duct was performed, followed by careful dissection around the sialolith to avoid injury to the surrounding tissues and prevent secondary duct stenosis. The stone was successfully removed and a seven-day course of antibiotics (amoxicillin + clavulanic acid, 1,000 mg twice a day) was prescribed postoperatively. The patient had an uneventful recovery and no further issues were reported at the two-month follow-up.

## Discussion

Sialolithiasis is a common disease of the salivary glands that appears more frequently in males with a wide range of age, usually between 30 and 60 years [2]. However, giant sialoliths, which are also called megaliths and are defined as stones with a maximum dimension greater than 15 mm, have rarely been reported [3]. A review of the recent literature revealed cases of large sialoliths of various sizes. Specifically, there were reports of sialoliths measuring 10 × 18 mm [4], 41 × 31 × 26 mm [5], and 35 × 25 × 15 mm [6]. However, there was no case of a stone in the submandibular duct with greater dimensions than those of our case (58 × 17 mm). To our knowledge, our case is the first report of a sialolith with an extremely unusual length diameter of almost 6 cm.

The exact mechanism leading to the pathogenesis of sialoliths remains unknown and various hypotheses have been proposed [7]. Some authors suggest that the existence of intracellular microcalculi, when excreted in the canal, may become a nidus for further calcification [8]. According to another theory, aliments, substances, or bacteria present within the oral cavity may be transferred into the salivary ducts and become the nidus for further calcification [9]. It is, therefore, implied that an initial organic nidus progressively grows by the deposition of layers of inorganic and organic substances [10]. The disease can be easily identified due to the obstruction of the gland by the presence of a sialolith [11]. The most common symptom is the swelling of the gland with associated pain, which increases while eating. Sialoliths can cause inflammation termed sialadenitis due to obstruction of the salivary duct and secondary bacterial infection. The latter is often a complication of sialolithiasis. When inflammation progresses, it can also result in abscess formation. In the case of a giant sialolith located within Wharton’s duct, the diagnosis is usually made by clinical examination, as the stone is palpable intraorally. The various differential diagnoses include inflammatory lesions, abscesses, and benign or malignant tumors, while a case with a sialolith mimicking an impacted tooth has also been described [12]. Therefore, in addition to history and clinical evaluation, further investigation with imaging such as CT and/or MRI scans may be helpful.

Therapeutic management includes conservative and/or surgical options [2]. In the case of sialolithiasis due to small stones, conservative management with adequate hydration, gland massage, and sialogogues to increase saliva production which can help in flushing the stone out of the duct, combined with antibiotic therapy if required, is usually sufficient. In contrast, for persistent cases or in the presence of large stones, surgical options such as interventional sialography, sialoendoscopy, lithotripsy, or surgery, including intraoral removal of the stone or excision of the submandibular gland, should be considered depending on the size and location of the sialolith and patient’s history [10].

## Conclusions

In our case, the sialolith had an extremely unusual diameter of almost 6 cm, and, to our knowledge, no similar case of a stone of this size in the submandibular duct has been reported in the literature. We demonstrated that intraoral removal of the stone can be a successful treatment modality for a patient with sialolithiasis secondary to a megalith, but in recurrent cases, a more aggressive approach with submandibular gland excision may be inevitable.
